# Changes in mandibular width and frontal-lower facial profile after orthognathic surgery using sagittal split ramus osteotomy with removal of internal bone interference in patients with class III skeletal malocclusion

**DOI:** 10.1186/s40902-024-00444-7

**Published:** 2024-09-10

**Authors:** In Jae Song, Min Seong Kang, Jung Han Lee, Eun Yeong Bae, Bok Joo Kim, Chul Hoon Kim, Jung Han Kim

**Affiliations:** 1 Department of Oral and Maxillofacial Surgery, College of Medicine, Dong-A University, Busan, Republic of Korea; 2 Department of Digital Solutions Technical Support, Megagen Implant Corporation, Seoul, Republic of Korea

**Keywords:** Class III skeletal malocclusion, SSRO, Bone interference, Mandibular width, Ramus inclination

## Abstract

**Background:**

The purpose of this study is to analyze changes in mandibular width and frontal view ramus inclination using cone beam CT in patients with skeletal class III malocclusion who underwent BSSRO, with the removal of bone interference between segments.

**Methods:**

For all 20 subjects, cone-beam CT imaging was performed prior to surgery (*T1*), immediately post-surgery (*T2*), and 6 months after surgery (*T3*). Reorientation was performed using R2GATE software (MegaGen, Seoul, Korea). The gonion and antegonial notch were used as reference points in the sagittal view, and the most lateral point of the condyle head was used as the reference point in the frontal view. All measurements were recorded in the frontal view.

**Results:**

Inter-gonial width decreased by 2.64 mm at T3-T2 (*P* < .001) and by 2.58 mm at T3-T1 (*P* < .05). Inter-antegonial width decreased by 1.75 mm at T3-T2 (*P* < .05) and by 3.5 mm at T3-T1 (*P* < .001). In the frontal view, the right ramus inclination based on the gonion increased by 2.07° at T3-T1 (*P* < .05). The left ramus inclination based on gonion increased by 2.45° at T2-T1 (*P* < .05) and by 3.94° at T3-T1 (*P* < .001). The right ramus inclination based on antegonial notch increased by 2.35° at T2-T1 (*P* < .05) and by 3.04° at T3-T1 (*P* < .01). The left ramus inclination based on antegonial notch increased by 2.73° at T2-T1 (*P* < .001) and by 3.18° at T3-T1 (*P* < .001).

**Conclusions:**

During bilateral sagittal split osteotomy, removing bone interference between the proximal and distal segments results in a reduction of postoperative mandibular width and an increase in frontal view ramus inclination.

## Background

Patients diagnosed with skeletal class III malocclusion show anterior placement of the mandible in relation to the maxilla. In cases of severe skeletal discrepancies, masticatory function may become inefficient, and pronunciation difficulties may arise during communication with others. Additionally, many patients are dissatisfied aesthetically due to facial elongation and a concave profile. To deal with these problems, orthognathic surgeries such as sagittal split ramus osteotomy (SSRO) and intraoral vertical ramus osteotomy (IVRO) are widely used.

When compared to IVRO, SSRO demonstrates various characteristics. Initially, following osteotomy, there is extensive contact between the cancellous bones of the proximal and distal segments, promoting active bone union after surgery. Additionally, the use of metal plates and screws for rigid fixation results in a decreased duration of intermaxillary fixation, thereby enabling early mouth opening.

However, there are instances in which mandibular width increases when compared to preoperative measurements following SSRO. This occurs because of bone interference between the distal and proximal segments. Bone interference, which is influenced by mandibular length, arch form, osteotomy techniques, and mandibular movement or rotation during surgery [1–4], can lead to an increase in mandibular width, potentially resulting in unsatisfactory aesthetic outcomes for patients wishing for a smaller and slimmer facial profile after surgery.

Various methods exist to reduce bone interference between the distal and proximal segments, thereby preventing an increase in mandibular width. Initially, IVRO may be performed rather than SSRO [1, 5]. Additionally, during SSRO, it is possible to perform short lingual osteotomy or distal segment ostectomy (DSO) [6–8]. When asymmetry is severe, one method involves angulating lingual osteotomy downwards on the deviated side while performing buccal osteotomy horizontally below the lingual osteotomy [5]. Finally, there is a method to directly remove the initial contact area between segments using surgical burrs or similar instruments.

The purpose of this study is to compare and analyze changes in mandibular width and frontal view ramus inclination using cone beam CT before surgery, immediately after surgery, and 6-month post-surgery in patients with skeletal class III malocclusion who underwent BSSRO, with the removal of bone interference between segments using surgical burrs.

## Materials and methods

### Subjects

In this study, 20 patients with completed growth and diagnosed with skeletal class III malocclusion underwent BSSRO at Dong-A University Hospital’s Department of Oral and Maxillofacial Surgery from January 1, 2020, to December 31, 2023. For all 20 subjects, cone-beam CT imaging was performed prior to surgery (T1), immediately post-surgery (T2), and 6 months after surgery (T3). Using Menton as a reference, chin deviation was categorized as symmetrical group if less than 3 mm and asymmetric group if 3 mm or more. Among 20 participants, there were 9 males with an average age of 22.9 years and 11 females with an average age of 22.8 years (Table 1). Patients who underwent mandibular angle osteotomy were excluded.

**Table 1:** Descriptive statistics

	**Group**		
**Variable**	**Symmetrical group (*n* = 9)**	**Asymmetrical group (*n* = 11)**	***p***
**Sex**			
Male	3 (33.3)	6 (54.5)	0.406^2^
Female	6 (66.7)	5 (45.5)	
**Age**	22.56 ± 3.84	23.09 ± 4.97	0.702^1^
**1-jaw/2-jaw**			
1-jaw	2 (22.2)	3 (27.3)	1.000^2^
2-jaw	7 (77.8)	8 (72.7)	
**Chin deviation before surgery**	0.80 ± 0.90	7.43 ± 3.17	< .001^1^
**Chin deviation after surgery**	2.32 ± 1.28	2.65 ± 1.64	0.970^1^
**Amount of setback**	6.39 ± 2.50	4.69 ± 4.62	.087^1^

Data are presented as mean ± SD

^1^*P*-values were derived by Mann–Whitney’s *U*-test

^2^*P*-values were derived from Fisher’s exact test. Shapiro–Wilk’s test was employed for test of normality assumption

### Surgery

**Fig. 1:**
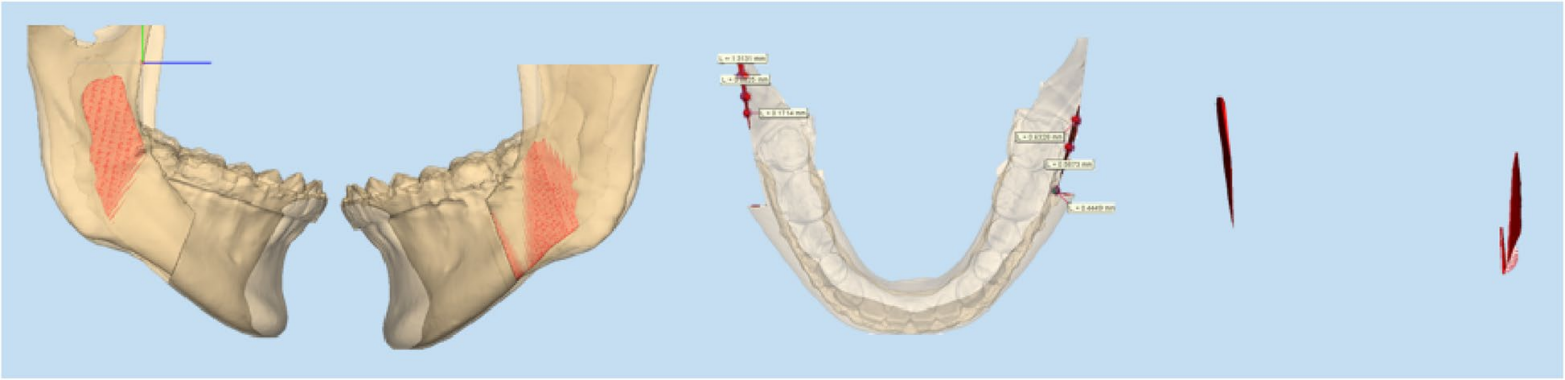
Preoperative bone interference 3D images acquired by the FaceGide software (MegaGen)

Among 20 patients, 15 underwent LeFort I and BSSRO, while 5 underwent only BSSRO. Orthognathic surgery was performed by an oral and maxillofacial surgeon for a period of 22 years. Before performing the surgery, FaceGide software (MegaGen, Seoul, Korea) is used to simulate osteotomy and fixation, assessing the bone interference area and amount between segments (Fig. 1). Before fixation with metal plates, surgical egg bur or round bur was used to remove bone interference between the proximal and distal segments.

### CBCT data

CBCT imaging (Green 16: Vatech Co., Seoul, Korea) was performed before surgery (T1), immediately after surgery (T2), and 6 months postoperatively (T3). The tube voltage was 94 kVp, the tube current was 8 mA, and the field of view was 160 × 90 mm. To minimize measurement errors related to head positioning during CBCT imaging, a manager with 6 years of experience in handling software at MegaGen used R2GATE software (MegaGen, Seoul, Korea) for reorientation before the orthognathic surgery. Reorientation was performed based on both the infra-orbital lower border in the frontal view and the Frankfort horizontal plane in the sagittal view. Additionally, the oral and maxillofacial surgery resident established the criteria for the reference point, and based on these criteria, the manager from MegaGen used FaceGide software (MegaGen, Seoul, Korea) to mark the reference point. The marked points were verified by the oral and maxillofacial surgery resident, and the final confirmation was given by the oral and maxillofacial surgery professor. 

### Reference point (Fig. 2)

**Fig. 2:**
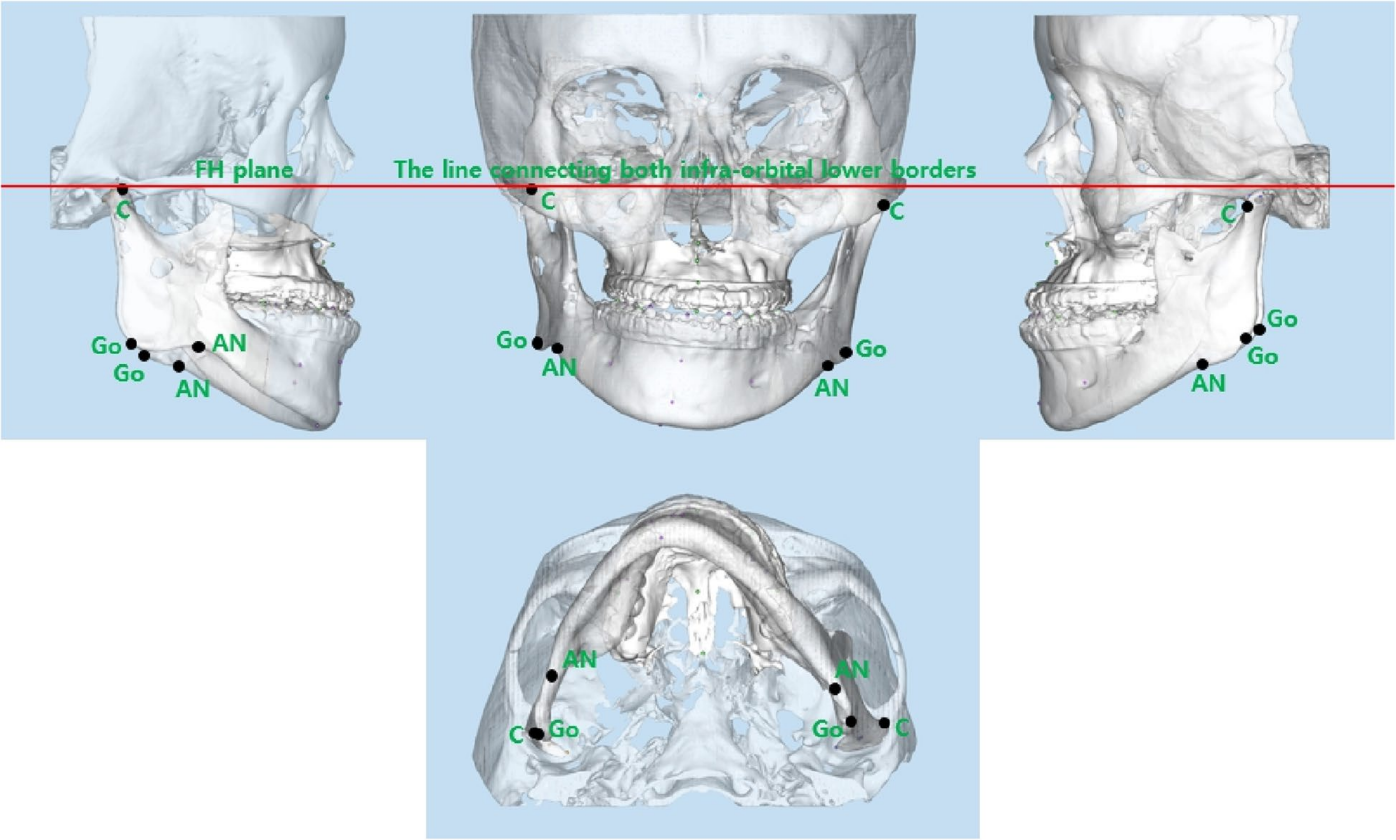
Reference points

- ▪ C: The most lateral point of condylar head on frontal view
- ▪ Go: Midpoint of posterior border of mandibular angle on sagittal view
- ▪ *AN*: The most antero-upper point on the antegonial notch on sagittal view

### Measured variables (Fig. 3)

**Fig. 3:**
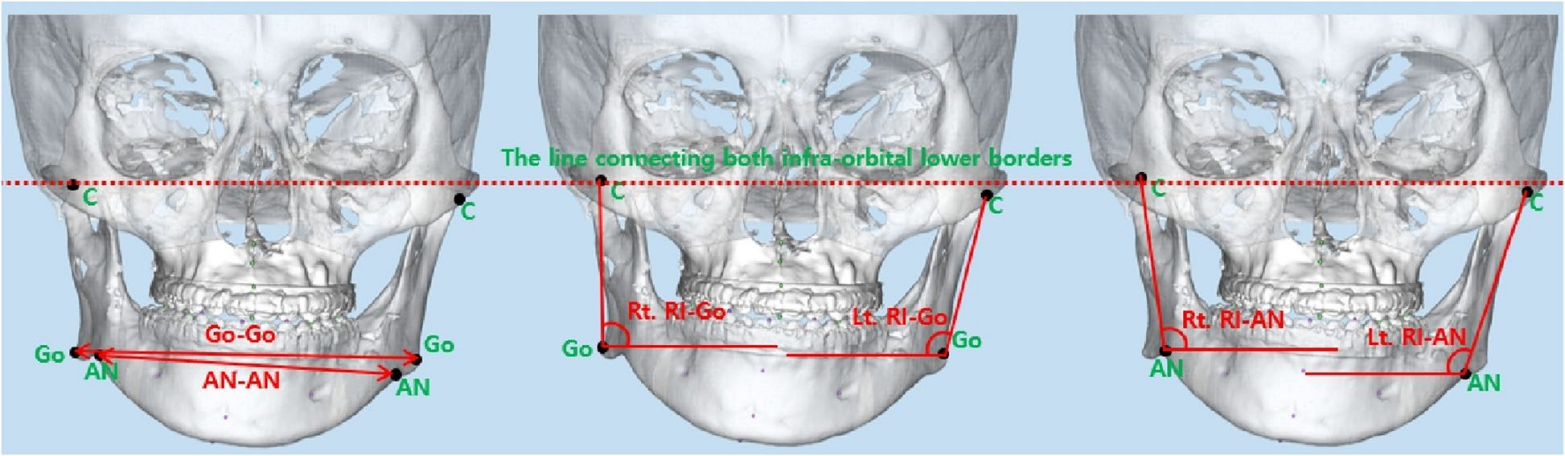
Measured variables

- ▪ Go-Go (inter-gonial width): Horizontal distance between the left and right Go
- ▪ *AN-AN* (inter-antegonial width): Horizontal distance between the left and right AN
- ▪ Rt. RI-Go: In frontal view, the angle between the line connecting the most lateral point of the condyle head to the right gonion and the line connecting both infraorbital lower borders
- ▪ Lt. RI-Go: In frontal view, the angle between the line connecting the most lateral point of the condyle head to the left gonion and the line connecting both infraorbital lower borders
- ▪ Rt. RI-AN: In frontal view, the angle between the line connecting the most lateral point of the condyle head to the right antegonial notch and the line connecting both infraorbital lower borders
- ▪ Lt. RI-AN: In frontal view, the angle between the line connecting the most lateral point of the condyle head to the left antegonial notch and the line connecting both infraorbital lower borders

### Statistical analysis

Variables were summarized by mean ± standard deviation (*SD*) for numeric data. Differences between two time-points were compared with paired *t*-test or Wilcoxon’s signed-rank test for numeric variables as appropriate. Group differences were tested using the independent *t*-test or Mann–Whitney *U*-test and analysis of variance or Kruskal–Wallis test for numeric data as appropriate. To check if its distribution is normal, we used Shapiro–Wilk’s test. Spearman’s correlation coefficients were used to assess the correlations among variables. Group differences were tested using the chi-squared test or Fisher’s exact for categorical data and for numeric data as appropriate. All statistical analyses were carried out using SPSS 26.0 (IBM Corp. Released 2019, IBM SPSS Statistics for Windows, Version 26.0, Armonk, NY: IBM Corp.), and *p*-values less than 0.05 were considered as statistically significant.

## Results

### Change in mandibular width

**Fig. 4:**
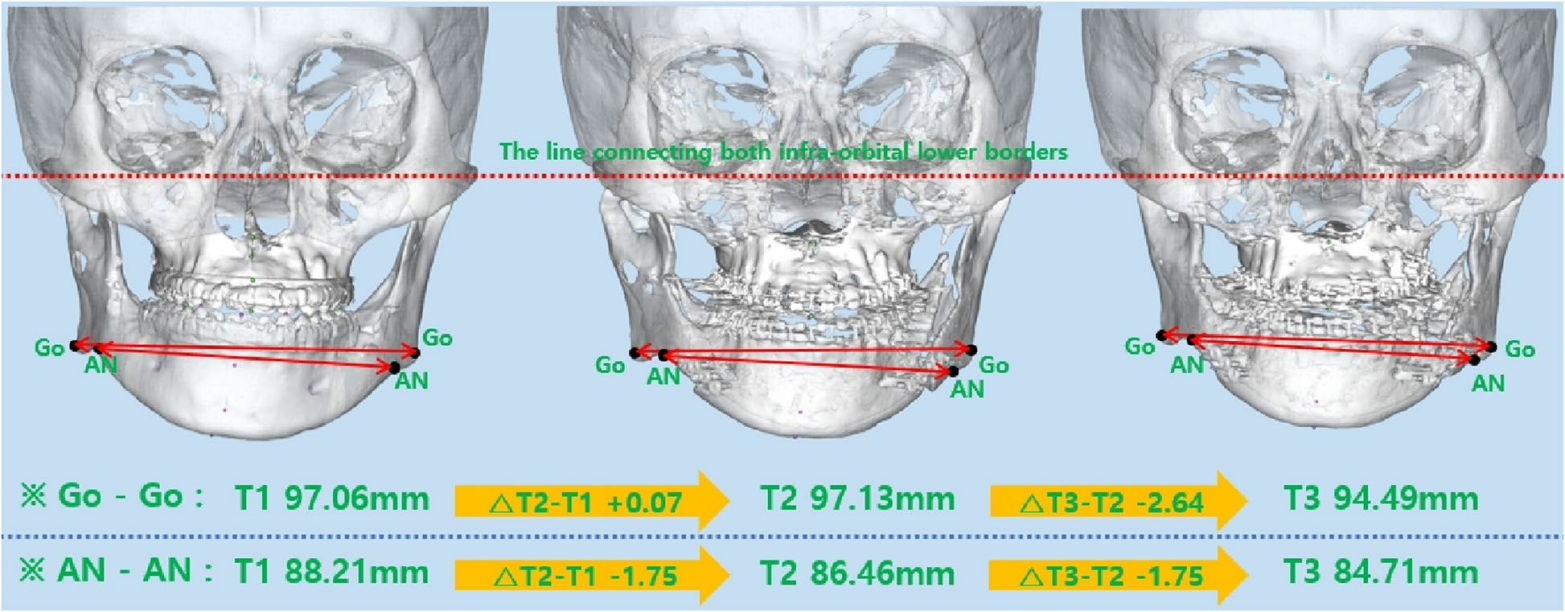
Changes in mandibular width

**Fig. 5:**
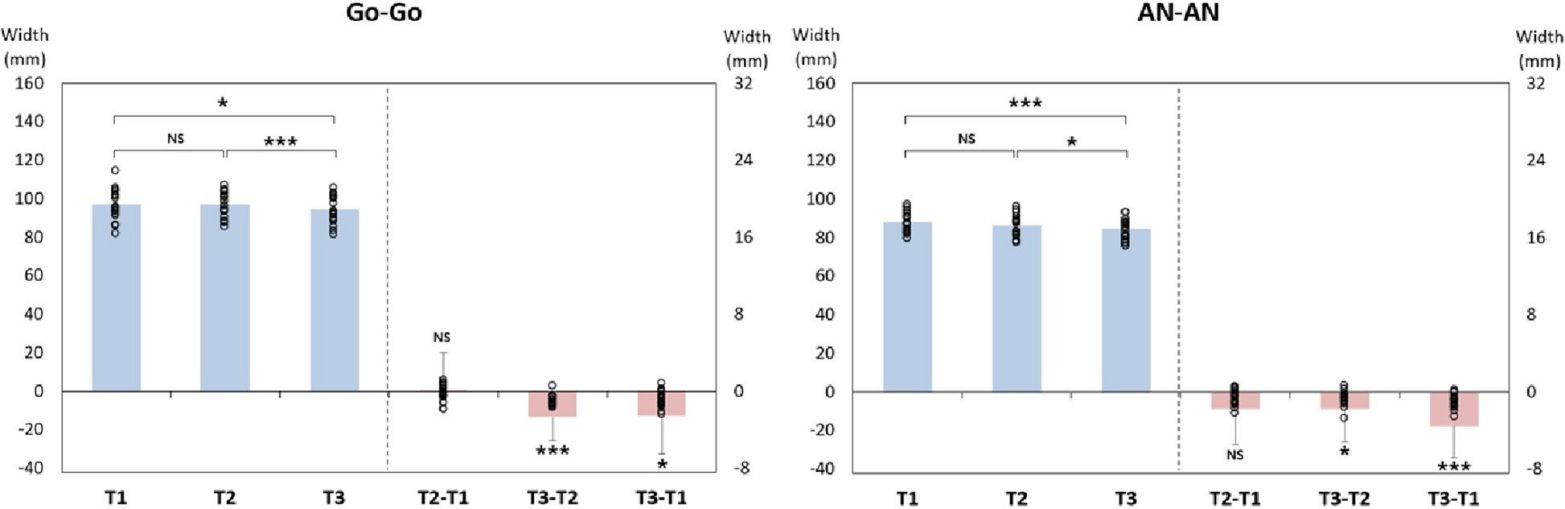
Bar graph for changes in mandibular width

A statistically significant decrease in Go-Go of 2.64 mm and 2.58 mm was observed at T3-T2 and T3-T1, respectively. AN-AN showed statistically significant reductions of 1.75 mm and 3.5 mm at T3-T2 and T3-T1, respectively (Figs. 4, 5 and Table 2).

**Table 2:** The average change in mandibular width

	**T1**	**T2**	**T3**	**T2-T1**		**T3-T2**		**T3-T1**	
Variable	Mean ± SD	Mean ± SD	Mean ± SD	Mean ± SD	***p***	Mean ± SD	***p***	Mean ± SD	***p***
Go-Go	97.06 ± 7.92	97.13 ± 6.29	94.49 ± 6.88	0.07 ± 3.99	0.940	− 2.64 ± 2.49	< .001	− 2.58 ± 3.97	.011
AN-AN	88.21 ± 4.84	86.46 ± 5.36	84.71 ± 5.10	− 1.75 ± 3.72	.059	− 1.75 ± 3.43	.014	− 3.50 ± 3.32	< .001

Data are presented as mean ± SD. *P*-values were derived from Wilcoxon signed-rank test. Shapiro–Wilk’s test was employed for test of normality assumption

### Change in frontal view ramus inclination

**Fig. 6:**
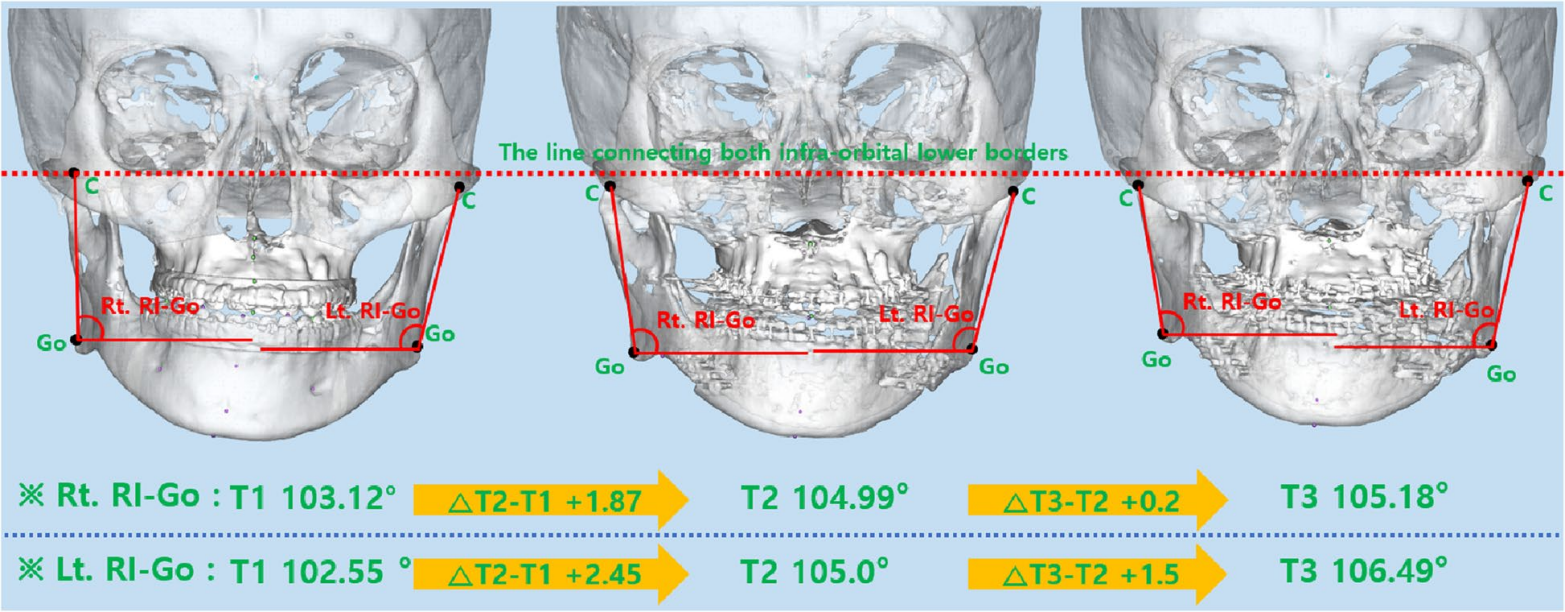
Changes in frontal view ramus inclination based on gonion

**Fig. 7:**
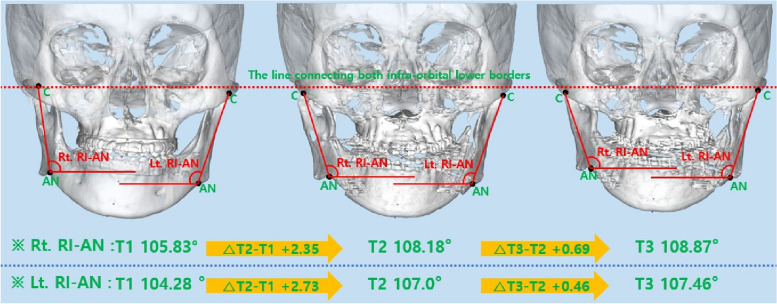
Changes in frontal view ramus inclination based on antegonial notch

**Fig. 8:**
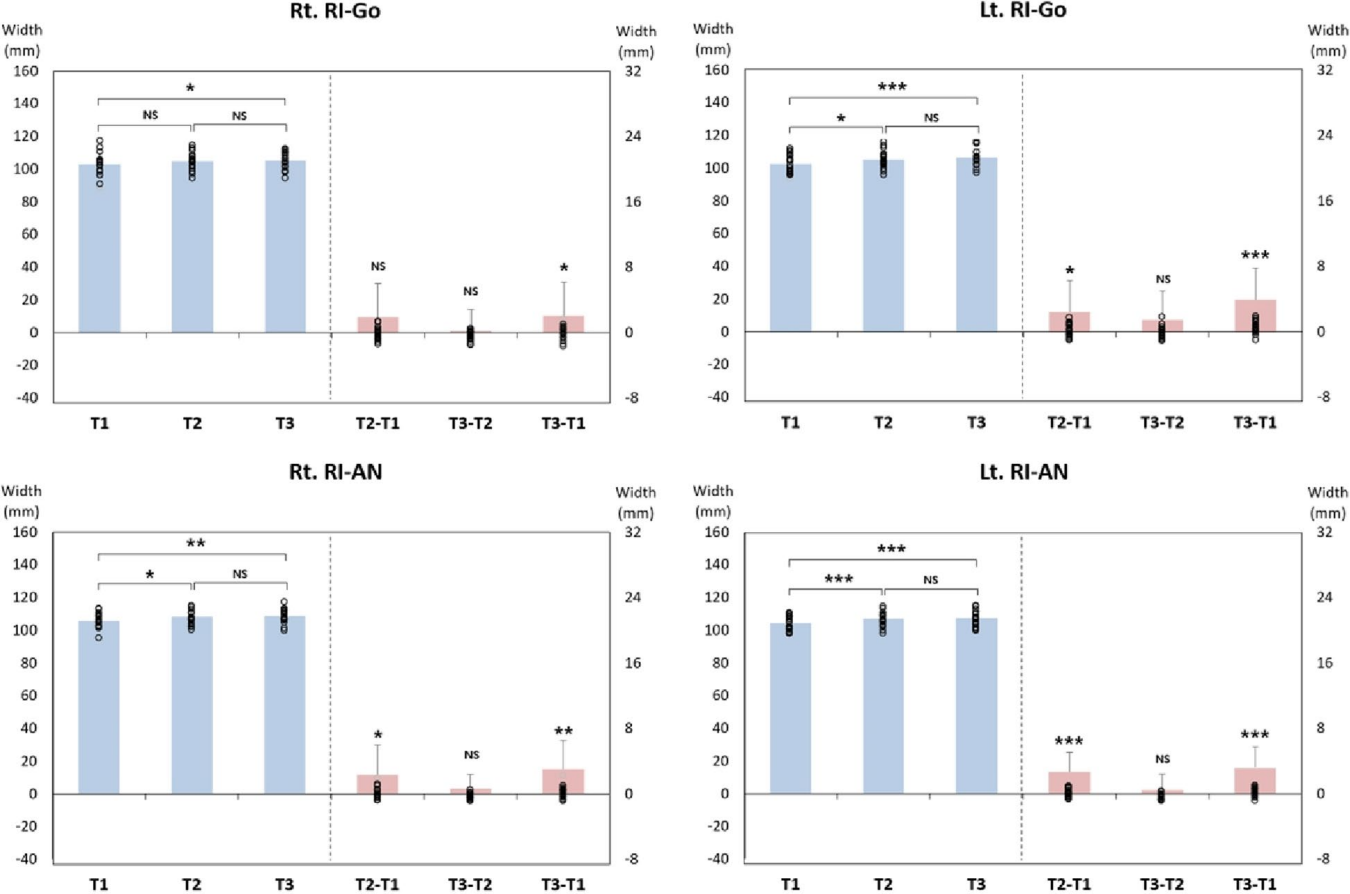
Bar graph for changes in frontal view ramus inclination

There was a statistically significant increase of 2.07° in Rt. RI-Go at T3-T1. There was a statistically significant increase of 2.45° and 3.94° in Lt. RI-Go at T2-T1 and T3-T1, respectively. At T2-T1 and T3-T1, there was a statistically significant increase of 2.35° and 3.04° observed in Rt. RI-AN. In each stage of T2-T1 and T3-T1, Lt. RI-AN statistically significantly increased by 2.73° and 3.18°, respectively (Figs. 6, 7, 8 and Table 3).

**Table 3:** The average change in ramus inclination

	**T1**	**T2**	**T3**	**T2-T1**		**T3-T2**		**T3-T1**	
Variable	Mean ± SD	Mean ± SD	Mean ± SD	Mean ± SD	***p***	Mean ± SD	***p***	Mean ± SD	**p**
Rt. RI-Go	103.12 ± 6.72	104.99 ± 5.13	105.18 ± 5.06	1.87 ± 4.10	0.100	0.20 ± 2.66	0.502	2.07 ± 4.12	.037
Lt. RI-Go	102.55 ± 5.39	105.00 ± 5.03	106.49 ± 5.48	2.45 ± 3.80	.020	1.50 ± 3.48	0.073	3.94 ± 3.85	< .001
Rt. RI-AN	105.83 ± 4.78	108.18 ± 3.96	108.87 ± 3.98	2.35 ± 3.62	.019	0.69 ± 1.75	0.126	3.04 ± 3.47	.002
Lt. RI-AN	104.28 ± 4.33	107.00 ± 4.10	107.46 ± 4.37	2.73 ± 2.33	< .001	0.46 ± 1.99	0.401	3.18 ± 2.51	< .001

Data are presented as mean ± SD. *P*-values were derived from Wilcoxon signed-rank test. Shapiro–Wilk’s test was employed for test of normality assumption

### Changes in mandibular width and ramus inclination by gender

At T2-T1, statistically significant increases were observed in Lt. RI-AN for males, while females showed statistically significant increases in Rt. RI-AN and Lt. RI-AN. At T3-T2, males showed no significance in all measurements, while females exhibited statistically significant decreases in Go-Go and AN-AN. Males showed a significant decrease in AN-AN at T3-T1, while there was a significant increase in Lt. RI-Go and Lt. RI-AN. Females showed a statistically significant decrease in AN-AN, while Lt. RI-Go, Rt. RI-AN, and Lt. RI-AN exhibited statistically significant increases (Table 4).

**Table 4:** Correlation in association with gender

	**T2-T1**				**T3-T2**				**T3-T1**			
	**Male (*n* = 9)**		**Female (*n* = 11)**		**Male (*n* = 9)**		**Female (*n* = 11)**		**Male (*n* = 9)**		**Female (*n* = 11)**	
**Variable**	**Mean ± SD**	***p***	**Mean ± SD**	***p***	**Mean ± SD**	***p***	**Mean ± SD**	***p***	**Mean ± SD**	***p***	**Mean ± SD**	***p***
**Go-Go**	− 1.86 ± 3.31	0.139	1.64 ± 3.92	0.248	− 1.60 ± 2.73	.086	− 3.50 ± 2.00	.003	− 3.45 ± 4.80	.066	− 1.86 ± 3.21	0.110
**AN-AN**	− 2.28 ± 3.69	.097	− 1.31 ± 3.86	0.328	− 1.35 ± 3.15	0.173	− 2.07 ± 3.76	.033	− 3.63 ± 2.50	.015	− 3.39 ± 3.98	.018
**Rt. RI-Go**	1.46 ± 3.30	0.374	2.21 ± 4.78	0.182	− 0.26 ± 2.64	0.953	0.56 ± 2.75	0.424	1.20 ± 3.77	0.374	2.77 ± 4.43	.075
**Lt. RI-Go**	3.14 ± 3.59	.051	1.87 ± 4.05	0.213	0.69 ± 2.48	0.374	2.15 ± 4.13	0.120	3.83 ± 3.97	.028	4.03 ± 3.95	.010
**Rt. RI-AN**	0.97 ± 2.52	0.407	3.48 ± 4.08	0.014	0.73 ± 1.31	0.155	0.65 ± 2.11	0.398	1.70 ± 2.63	0.109	4.13 ± 3.80	.004
**Lt. RI-AN**	3.02 ± 3.03	.038	2.48 ± 1.68	.003	− 0.16 ± 1.81	0.859	0.95 ± 2.08	0.286	2.87 ± 2.81	.028	3.44 ± 2.35	.007

Data are presented as mean ± SD. *P*-values were derived from Wilcoxon signed-rank test. Shapiro–Wilk’s test was employed for test of normality assumption

### The correlation between mandibular setback, mandibular width, and ramus inclination

When the setback amount is less than 5 mm at T2-T1, Lt. RI-AN showed a significant increase of 2.29°, while for setback amounts between 5 and 9 mm, Lt. RI-Go increased by 4.9°, and Lt. RI-AN increased by 4.17°, respectively. When the setback amount is less than 5 mm at the T3-T2, a significant decrease of 2.88 mm was observed in Go-Go. When the setback was between 5 and 9 mm, Go-Go showed a significant reduction of 2.63 mm, while AN-AN decreased by 1.56 mm, and a significant increase of 1.20° was observed in Lt. RI-AN. When the setback amount was less than 5 mm at T3-T1, Go-Go decreased by 2.54 mm, and AN-AN decreased by 2.74 mm, while Lt. RI-Go increased significantly by 3.27°, Rt. RI-AN by 3.33°, and Lt. RI-AN by 2.42°. When the setback was between 5 and 9 mm, Go-Go showed a significant reduction of 3.82 mm, AN-AN decreased by 3.79 mm, Lt. RI-Go increased significantly by 6.35°, and Lt. RI-AN increased significantly by 5.37° (Table 5).

**Table 5:** Correlation in association with the setback amount

	**T2-T1**						**T3-T2**						**T3-T1**					
	** < 5 mm (*n* = 10)**		**5 ~ 9 mm (*n* = 6)**		** > 9 mm (*n* = 4)**		** < 5 mm (*n* = 10)**		**5 ~ 9 mm (*n* = 6)**		** > 9 mm (*n* = 4)**		** < 5 mm (*n* = 10)**		**5 ~ 9 mm (*n* = 6)**		** > 9 mm (*n* = 4)**	
**Variable**	**Mean ± SD**	***p***	**Mean ± SD**	***p***	**Mean ± SD**	***p***	**Mean ± SD**	***p***	**Mean ± SD**	***p***	**Mean ± SD**	***p***	**Mean ± SD**	**p**	**Mean ± SD**	**p**	**Mean ± SD**	***p***
**Go-Go**	0.34 ± 4.72	0.878	− 1.20 ± 3.77	0.600	1.29 ± 2.19	0.465	− 2.88 ± 2.09	.005	− 2.63 ± 1.88	.028	− 2.08 ± 4.42	0.465	− 2.54 ± 3.66	.047	− 3.82 ± 3.71	.046	− 0.79 ± 5.45	0.715
**AN-AN**	− 0.80 ± 3.83	0.359	− 2.23 ± 4.20	0.249	− 3.41 ± 2.66	.068	− 1.94 ± 4.76	0.203	− 1.56 ± 1.38	.046	− 1.54 ± 1.75	0.144	− 2.74 ± 3.11	.028	− 3.79 ± 4.38	.046	− 4.95 ± 1.92	.068
**Rt. RI-Go**	1.56 ± 3.84	0.285	2.35 ± 4.74	0.345	1.93 ± 4.90	0.461	0.43 ± 1.87	0.333	0.67 ± 2.43	0.600	− 1.10 ± 4.65	0.465	1.99 ± 4.60	0.169	3.02 ± 3.84	0.173	0.83 ± 3.93	0.715
**Lt. RI-Go**	1.43 ± 3.73	0.386	4.90 ± 3.58	.028	1.30 ± 3.39	0.465	1.84 ± 4.27	0.284	1.45 ± 1.78	0.116	0.70 ± 3.94	0.715	3.27 ± 3.33	.017	6.35 ± 3.98	.028	2.00 ± 4.03	0.273
**Rt. RI-AN**	1.94 ± 4.11	0.308	2.75 ± 3.67	0.141	2.78 ± 2.97	.068	1.39 ± 1.79	.059	− 0.17 ± 1.44	0.600	0.20 ± 1.70	0.715	3.33 ± 4.30	.037	2.58 ± 3.31	0.173	2.98 ± 1.30	.068
**Lt. RI-AN**	2.29 ± 2.44	.011	4.17 ± 1.83	.028	1.65 ± 2.13	0.273	0.13 ± 2.17	0.683	1.20 ± 1.22	.028	0.15 ± 2.63	0.715	2.42 ± 2.24	.011	5.37 ± 1.48	.028	1.80 ± 2.68	0.144

Data are presented as mean ± SD. *P*-values were derived from Wilcoxon signed-rank test. Shapiro–Wilk’s test was employed for test of normality assumption

### The correlation between asymmetry, mandibular width, and ramus inclination

At the T2-T1, when asymmetry was less than 3 mm, Lt. RI-AN significantly increased by 2.58°, and when asymmetry ranged from 3 to 5 mm, Lt. RI-Go increased significantly by 3.56°, and Lt. RI-AN increased by 3.2°. When exceeding 5 mm, Lt. RI-AN significantly increased by 2.29°. At T3-T2, when asymmetry was less than 3 mm, there was a significant decrease in Go-Go by 2.62 mm. When asymmetry was 3–5 mm, Go-Go decreased significantly by 2.88 mm, while Lt. RI-Go increased significantly by 2.03°. When exceeding 5 mm, the AN-AN significantly decreased by 1.48 mm. At the T3-T1, when asymmetry was less than 3 mm, AN-AN significantly decreased by 5.92 mm, while Lt. RI-AN increased by 3.64°. With asymmetry between 3 and 5 mm, Go-Go significantly decreased by 3.64 mm, Lt. RI-Go increased by 5.59°, and Lt. RI-AN increased by 4.01°. When asymmetry exceeded 5 mm, Rt. RI-AN significantly increased by 4.73° (Table 6).

**Table 6:** Correlation in association with asymmetry

	**T2-T1**						**T3-T2**						**T3-T1**					
	** < 3 mm (*n* = 5)**		**3 ~ 5 mm (*n* = 8)**		** > 5 mm (*n* = 7)**		** < 3 mm (*n* = 5)**		**3 ~ 5 mm (*n* = 8)**		** > 5 mm (*n* = 7)**		** < 3 mm (*n* = 5)**		**3 ~ 5 mm (*n* = 8)**		** > 5 mm (*n* = 7)**	
**Variable**	**Mean ± SD**	***p***	**Mean ± SD**	***p***	**Mean ± SD**	**p**	**Mean ± SD**	**p**	**Mean ± SD**	***p***	**Mean ± SD**	***p***	**Mean ± SD**	***p***	**Mean ± SD**	***p***	**Mean ± SD**	***p***
**Go-Go**	0.75 ± 2.65	0.893	− 0.76 ± 3.80	0.484	0.53 ± 5.22	0.735	− 2.62 ± 2.55	.043	− 2.88 ± 1.81	.012	− 2.39 ± 3.36	.091	− 1.88 ± 1.62	.080	− 3.64 ± 3.38	.025	− 1.86 ± 5.69	0.499
**AN-AN**	− 2.22 ± 3.67	0.225	− 1.27 ± 3.64	0.327	− 1.96 ± 4.33	0.310	− 3.69 ± 4.91	.080	− 0.76 ± 3.54	0.484	− 1.48 ± 1.45	.028	− 5.92 ± 1.60	.043	− 2.03 ± 2.62	.058	− 3.44 ± 4.17	.063
**Rt. RI-Go**	2.48 ± 4.37	0.225	1.16 ± 4.03	0.575	2.24 ± 4.51	0.236	0.06 ± 3.00	0.686	0.66 ± 2.83	0.400	− 0.24 ± 2.55	0.735	2.54 ± 2.58	0.138	1.83 ± 3.26	0.123	2.00 ± 6.05	0.398
**Lt. RI-Go**	2.12 ± 3.86	0.345	3.56 ± 3.91	.012	1.40 ± 3.87	0.499	− 0.66 ± 2.75	0.500	2.03 ± 2.08	.035	2.43 ± 4.79	0.237	1.46 ± 1.28	.080	5.59 ± 3.15	.012	3.83 ± 5.06	.091
**Rt. RI-AN**	2.76 ± 3.06	.080	0.86 ± 2.57	0.528	3.76 ± 4.70	.063	0.78 ± 2.83	0.686	0.38 ± 1.33	0.401	0.97 ± 1.44	0.128	3.54 ± 2.70	.078	1.24 ± 2.38	0.208	4.73 ± 4.33	.018
**Lt. RI-AN**	2.58 ± 2.03	.043	3.20 ± 3.09	.030	2.29 ± 1.65	.028	1.06 ± 2.33	0.500	0.81 ± 2.23	0.401	− 0.39 ± 1.35	0.499	3.64 ± 1.57	.043	4.01 ± 2.65	.017	1.90 ± 2.67	0.116

Data are presented as mean ± SD. *P*-values were derived from Wilcoxon signed-rank test. Shapiro–Wilk’s test was employed for test of normality assumption

## Discussion

When skeletal class III malocclusion is present, patients may have difficulties with chewing, pronunciation, and aesthetic appearance. To resolve these issues, orthognathic surgeries, such as SSRO and IVRO, are frequently used. Since Trauner and Obwegeser [9] introduced it in 1957, SSRO has been extensively used in orthognathic surgery, presenting various characteristics. Healing occurs rapidly due to extensive cancellous bone contact between the proximal and distal segments, enabling early mouth opening. However, surgical time is extended, and there is a possibility of inferior alveolar nerve damage. Particularly, if bone interference or gaps between the proximal and distal segments are not appropriately addressed during fixation with metal plates, postoperative mandibular width may widen compared to preoperative width. This may lead to aesthetically dissatisfying outcomes for patients who desire a small and slender facial profile after surgery.

There are several methods to remove bone interference. Modifying the osteotomy technique offers a means to decrease interference between the proximal and distal segments. According to Ueki and Yoshida [1, 5], IVRO can create a shorter distal segment compared to SSRO, while according to Hunsuck and Wolford [6, 7], performing short lingual osteotomy during SSRO can result in a shorter distal segment than conventional SSRO. Creating such a short distal segment and moving it to the planned position reduces bone interference with the proximal segment compared to conventional SSRO. Ellis [8] proposed a strategy that entails osteotomizing the distal segment behind the terminal molar to induce a greenstick fracture. This method aims to eliminate premature contacts in all areas, enabling passive contact between segments and reducing the possibility of condylar displacement from the mandibular fossa. According to Yoshida [5], lingual osteotomy on the deviated side should be angled downward, while buccal osteotomy should be conducted horizontally below the lingual osteotomy. This creates a triangular space, preventing bone interference when performing fixation. Another method involves directly removing the area of initial contact between segments causing interference, using surgical burrs. The direct removal method is challenging due to limited visibility and the risk of nerve damage, and it is time-consuming. However, with extensive bone contact between segments, rapid bone union can be achieved, leading to early postoperative stability.

To address bone interference between the proximal and distal segments, this study directly removed the initially contacted area. The virtual osteotomy and fixation are simulated preoperatively using FaceGide software (MegaGen, Seoul, Korea) to assess the area and amount of bone interference between segments. Following MMF in surgery, confirm passive contact between segments before applying metal plate fixation and clinically determine the necessary areas and amounts of bone interference to be removed. When a large amount of removal is required, a straight low handpiece and surgical egg bur are used; otherwise, a round bur is used. Remove bone interference and appropriately bend the metal plate for fixation. Before completing the surgery, repeated checks of occlusion and jaw movement are conducted to confirm changes in the position of the condyle after fixation.

Previously, two-dimensional cephalometric analysis was used to evaluate facial profiles before and after surgery. Consistently replicating the patient’s head position during radiographic imaging is challenging. Differences in magnification due to focal distance can cause image distortion, overlap, ghosting, and artifacts, making it difficult to identify reference points. Therefore, two-dimensional cephalometric analysis has limitations in evaluating facial changes pre- and post-surgery. These limitations can be overcome with CBCT. By constructing three-dimensional images of the facial bones, one can easily and intuitively comprehend anatomical information that reduces magnification or distortion.

Reorientation is crucial for comparing and analyzing preoperative and postoperative changes using CBCT. According to Kim [10], the altered head position during CBCT imaging had an impact on the coordinates of anatomical landmarks, but accurate alignment was achieved through reorientation using image analysis software. In this study, reorientation was performed using R2GATE software (MegaGen, Seoul, Korea). Reorientation in the frontal view was based on a line connecting the bilateral infraorbital lower borders, while in the sagittal view, it was referenced to the Frankfort horizontal plane. The gonion and antegonial notch were used as reference points in the sagittal view, and the lateral point of the condyle head was used as the reference point in the frontal view. All measurements were recorded in the frontal view.

This study compared the inter-gonial width (Go-Go) and inter-antegonial width (AN-AN) in 20 skeletal class III patients who underwent BSSRO before and after surgery. At T2-T1, there were no statistically significant changes in Go-Go and AN-AN. At T3-T2, Go-Go decreased by 2.64 mm, and AN-AN decreased by 1.75 mm. At T3-T1, Go-Go decreased by 2.58 mm, and AN-AN decreased by 3.5 mm. According to Choi et al. [11], Go-Go increased by 3.6 mm at T2-T1, decreased by 1.6 mm at T3-T2, and increased by 2.1 mm at T3-T1. Moroi et al. [12] categorized patients into a symmetry group when the maxillomandibular midline angle was below 2.5° and into an asymmetry group otherwise. In the symmetry group, the mandibular width was measured at 95.2 mm before surgery, 99.8 mm 1 week after, and 99.7 mm a year later. In the asymmetry group, measurements were 95.8 mm before surgery, 99.7 mm 1 week after, and 98.4 mm 1 year later. In their study, Chen et al. [13] divided the subjects into two categories based on pogonion, one for setbacks of 8 mm or less and the other for setbacks over 8 mm. For the group with a setback of 8 mm or less, the Go-Go increased by 0.3 mm at T2-T1, decreased by 1.4 mm at T3-T2, and decreased by 1.1 mm at T3-T1; however, these changes were not statistically significant. In groups with a setback greater than 8 mm, T2-T1 increased by 4.6 mm, T3-T2 decreased by 0.7 mm, and T3-T1 increased by 4.0 mm. According to Kim et al. [14], setbacks of 2 mm or less were classified as symmetry group, while those exceeding 2 mm were classified as asymmetry group. In the symmetry group, Go-Go increased by 2.91 mm at T2-T1, decreased by 1.62 mm at T3-T2, and increased by 1.29 mm at T3-T1. In the asymmetry group, Go-Go increased by 2.47 mm at T2-T1, decreased by 1.32 mm at T3-T2, and increased by 1.15 mm at T3-T1. In this study, the inter-gonial width and inter-antegonial width remained mostly unchanged from T2 to T1. If condylar lateral displacement occurs as a result of bony interference and fixation, it is possible for both inter-gonial width and inter-antegonial width to increase. However, if bone interference is sufficiently removed to create passive contact between segments and fixation is applied, lateral displacement of the condyle can be minimized. Consequently, as the proximal segment rotates around the condyle, the anterior part of the proximal segment moves medially, which could lead to a decrease in inter-antegonial width without increasing inter-gonial width. At T3-T2, it is thought that this is a result of bone remodeling between segments and in the gonial region after surgery.

In this study, we analyzed changes in ramus inclination as observed in the frontal view to assess the lower facial profile. The change in mandibular width at T2-T1 was not significant, but ramus inclination (both RI-Go and RI-AN) significantly increased in all cases except Rt. RI-Go. In the frontal view, ramus inclination is affected by changes in the most lateral point of the condylar head, gonion, and antegonial notch. If the condylar position remains stable, a decrease in mandibular width results in an increase in ramus inclination. In this study, the decrease in mandibular width was greater at T3-T2 than at T2-T1, indicating that ramus inclination should have increased significantly at T3-T2. However, there was a greater increase in ramus inclination at T2-T1. It is believed that this occurred because the condyle was slightly displaced laterally in the mandibular fossa immediately after surgery, and it was repositioned after 6 months.

In this study, there was no statistical significance observed in the amount of setback and changes in mandibular width. According to by Kim [14], despite significant mandibular setback, there was no proportional change in inter-gonial width. When the mandible is set back, it is thought that direct bone interference between segments decreases as the distal segment shifts to the broader posterior area of the mandible. Therefore, the mandibular width will decrease after setback and fixation. However, if the amount of setback is significant, a large gap may form between the distal and proximal segments. If fixation is performed without considering the gap, the condyle may shift laterally within the mandibular fossa. It is thought to be a factor that may lead to an increase in mandibular width after surgery.

In this study, there was no statistically significant difference in asymmetry and mandibular width changes. According to Kim [14], in cases of asymmetry, a substantial increase in unilateral gonial width was observed on the deviated side compared to the non-deviated side, although it was not statistically significant. However, it seems unreasonable to consider the correlation between pre- and postoperative mandibular width solely on the basis of the degree of asymmetry. When canting is not present, asymmetry can be corrected by yawing the segment around the *Y*-axis or moving it bodily. When the mandible is moved bodily, bone interference occurs on the non-deviated side, whereas yawing mainly causes bone interference on the deviated side. In the presence of canting, rolling the segment about the *Z*-axis for correction leads to more bone interference on the non-deviated side than on the deviated side. If asymmetry remains after correcting canting, the segment can be moved bodily to the non-deviated side or yawed around the *Y*-axis. When moved bodily to the non-deviated side, bone interference on the non-deviated side increases. Yawing causes increased bone interference on the deviated side. Therefore, the correlation between asymmetry and mandibular width should be considered, taking into account the degree of asymmetry and the movement and rotation of the distal segment. Similar to the previous study, this study found no significant relationship between asymmetry and mandibular width; however, the precise removal of bone interference between the proximal and distal segments with a surgical burr may have influenced these results.

There are some challenges and limitations encountered in this study. First, we identified anatomical landmarks that remain unchanged before and after surgery. According to Pan [15], the mandibular angle may be included in the surgical area, and considerable bone remodeling is observed in the gonial region. Therefore, in this study, reference points were defined on the sagittal view of CBCT, where gonion and antegonial notch were most readily observed, and measurements were conducted; however, defining these points was difficult in certain cases. Secondly, there were limitations in considering changes in condylar morphology and position after surgery. In this study, the most lateral point of the condyle head was chosen as the reference point using FaceGide software (MegaGen, Seoul, Korea) before and after surgery. However, according to Lin [16], the morphology of the condyle can change due to remodeling after SSRO. Additionally, sagging and rotation of the condyle may occur after surgery. These changes in condyle morphology and position can alter the most lateral point of the condylar head before and after surgery. However, this research did not take into account the changes in the condyle before and after surgery. Third, the amount of setback, asymmetry, and canting were not categorized by range; thus, it was not possible to analyze changes in mandibular width before and after surgery based on these classifications. The movement and rotation of the distal segment vary according to each variable, leading to bone interference between segments. Therefore, it is believed that more research is needed to understand the correlation between changes in mandibular width and ramus inclination and various variables, by categorizing these variables.

## Conclusion

In a study involving 20 patients who underwent BSSRO for skeletal class III malocclusion, changes in mandibular width and ramus inclination were assessed using 3D CT scans at preoperative (T1), immediate postoperative (T2), and 6-month postoperative (T3) stages. Removing bone interference between the proximal and distal segments during bilateral sagittal split osteotomy reduces postoperative mandibular width and increases ramus inclination when viewed from the front.

## Data Availability

The datasets used and/or analyzed during the current study are available from the corresponding author on reasonable request.

## Abbreviations

BSSRO: Bilateral sagittal split ramus osteotomy
SSRO: Sagittal split ramus osteotomy
IVRO: Intraoral vertical ramus osteotomy
DSO: Distal segment ostectomy
CBCT: Cone-beam computed tomography
CT: Computed tomography
